# Apararenone in patients with diabetic nephropathy: results of a randomized, double-blind, placebo-controlled phase 2 dose–response study and open-label extension study

**DOI:** 10.1007/s10157-020-01963-z

**Published:** 2020-09-24

**Authors:** Takashi Wada, Masaya Inagaki, Toru Yoshinari, Ryuji Terata, Naoko Totsuka, Miki Gotou, Gaia Hashimoto

**Affiliations:** 1 Department of Nephrology and Laboratory Medicine, Kanazawa University, Kanazawa, Japan; 2 Data Science Department, Ikuyaku. Integrated Value Development Division, Mitsubishi Tanabe Pharma Corporation, Tokyo, Japan; 3 Clinical Research and Development II Department, Ikuyaku. Integrated Value Development Division, Mitsubishi Tanabe Pharma Corporation, 17-10, Nihonbashi-Koamicho, Chuo-ku, Tokyo, 103-8405 Japan

**Keywords:** Diabetic nephropathy, Diabetic kidney disease, Dose-finding study, Fibrosis, Mineralocorticoid receptor antagonists, Apararenone

## Abstract

**Background:**

We investigated the efficacy and safety of apararenone (MT-3995), a non-steroidal compound with mineralocorticoid receptor agonist activity, in patients with stage 2 diabetic nephropathy (DN).

**Methods:**

The study had two parts: a dose–response, parallel-group, randomized, double-blind, placebo-controlled, multicenter, phase 2, 24-week study and an open-label, uncontrolled, 28-week extension study. Primary and secondary endpoints were the 24-week percent change from baseline in urine albumin to creatine ratio (UACR) and 24- and 52-week UACR remission rates. Safety parameters were changes from baseline in estimated glomerular filtration rate (eGFR) and serum potassium at 24 and 52 weeks, and incidences of adverse events (AEs) and adverse drug reactions (ADRs).

**Results:**

In the dose–response period, 73 patients received placebo and 73, 74, and 73 received apararenone 2.5 mg, 5 mg, and 10 mg, respectively. As a percentage of baseline, mean UACR decreased to 62.9%, 50.8%, and 46.5% in the 2.5 mg, 5 mg, and 10 mg apararenone groups, respectively, at week 24 (placebo: 113.7% at week 24; all *P* < 0.001 vs placebo). UACR remission rates at week 24 were 0.0%, 7.8%, 29.0%, and 28.1% in the placebo and apararenone 2.5 mg, 5 mg, and 10 mg groups, respectively. eGFR tended to decrease and serum potassium tended to increase, but these events were not clinically significant. AE incidence increased with dose while ADR incidence did not.

**Conclusion:**

The UACR-lowering effect of apararenone administered once daily for 24 weeks in patients with stage 2 DN was confirmed, and the 52-week administration was safe and tolerable.

**Clinical trial registration:**

NCT02517320 (dose–response study) and NCT02676401 (extension study)

**Electronic supplementary material:**

The online version of this article (10.1007/s10157-020-01963-z) contains supplementary material, which is available to authorized users.

## Introduction

Diabetic nephropathy (DN)/diabetic kidney disease (DKD) is the leading cause of chronic kidney disease worldwide [1]. Since 1998, DN is the main primary disease attributable to the introduction of maintenance dialysis in Japan. DN patients accounted for 42.5% of the total number of patients on dialysis in 2017 [2]. The 5-year survival rate of patients on dialysis in 2010 was 60.8% [2]. Thus, it is important to diagnose and treat diabetes patients with early-stage DN to inhibit disease progression, improve patient prognosis and quality of life, and decrease the economic burden of the disease.

Microalbuminuria occurs during the early stages of DN and a urine albumin to creatine ratio (UACR) of 30–299 mg/gCr is indicative of early-stage nephropathy [3]. Microalbuminuria is associated with the initiation of dialysis, kidney transplantation, reduced estimated glomerular filtration rate (eGFR), and risk of progression to overt nephropathy. As the urine albumin level increases, the incidence of cardiovascular events increases [4] and the prognosis for renal function worsens [5]. Thus, early therapeutic intervention is desirable.

Results of large-scale clinical studies [6] revealed that overactivity of the renin–angiotensin–aldosterone system was involved in the renal function deterioration of patients with albuminuria/proteinuria who had DN or chronic kidney disease stage G3 or G4. Clinical studies reported that angiotensin-converting enzyme inhibitors (ACE-I) and angiotensin II receptor blockers (ARBs) inhibit increases in urine albumin/urine protein and progression of renal dysfunction in early-stage nephropathy [7, 8]. Treatment guidelines recommend the use of these drugs for renal protection [2, 9]. In Japan, the ARB losartan potassium is the only drug indicated for DN. However, it is only indicated for patients with hypertension and proteinuria (UACR of ≥ 300 mg/gCr, corresponding to overt nephropathy stage). Thus, no therapeutic drug is currently available for patients with early-stage nephropathy.

Mineralocorticoid receptors (MRs) are present in podocytes, mesangial cells, vascular endothelial cells, and fibroblasts of the kidney, suggesting that MR antagonists (MRAs) may have renal-protective effects through fibrosis prevention [10, 11]. Apararenone (MT-3995), discovered by Mitsubishi Tanabe Pharma Corporation, Tokyo, Japan, is a non-steroidal compound with highly selective MRA activity. Nonclinical studies suggest that apararenone may have superior efficacy and safety profiles than similar drugs (data on file). Clinical studies on other non-steroidal MRAs such as finerenone [12] and esaxerenone [13] have confirmed the UACR-lowering effect of these drugs, but those studies were limited by short treatment periods. We investigated the efficacy of apararenone versus placebo using the UACR in first morning void urine samples as an index of microalbuminuria in patients with stage 2 DN (early-stage nephropathy), dose–response to apararenone based on UACR (main efficacy index), and efficacy and safety of long-term treatment (52 weeks) with apararenone.

## Materials and methods

### Study design

This study had two parts (Supplementary Figure 1): a dose–response study and an extension study. The dose–response study was a parallel-group, randomized, double-blind, placebo-controlled, multicenter phase 2 study of type 2 diabetes patients with stage 2 DN conducted from 24 July 2015 to 7 January 2017 in Japan.

The dose–response study had a run-in period of 4 weeks, followed by a treatment period of 24 weeks and a follow-up period of 8 weeks. The 8-week follow-up period did not apply to patients in the extension study. Patients were randomly assigned to receive apararenone 2.5 mg, 5 mg, or 10 mg or placebo by a permuted block method and stratification by presence or absence of concomitant use of ACE-I or ARB and UACR (first morning void urine) 2 weeks before randomization.

Patients from the dose–response study who met the criteria entered the extension study. The extension study comprised a 28-week (starting from randomization until the day of evaluation), parallel-group, randomized, open-label, uncontrolled, multicenter phase 2 extension study conducted from 4 January 2016 to 29 August 2017, including an 8-week follow-up period. Other details of the extension study are provided in the Supplementary Text.

### Treatment

During the dose–response study, patients took apararenone 2.5 mg, 5 mg, or 10 mg or placebo orally once a day (after breakfast) for 24 weeks (from randomization to the day before the evaluation), and those in the extension study took apararenone 2.5 mg, 5 mg, and 10 mg orally once a day for 28 weeks. The prohibited concomitant and other treatment details are summarized in the Supplementary Text.

### Participants

Patients enrolled in the dose–response study were Japanese men or women aged 20–75 years, with type 2 diabetes mellitus according to the diagnostic criteria of the Japan Diabetes Society [9]; glycated hemoglobin ≤ 10.5%; eGFR ≥ 30 mL/min/1.73 m^2^ (Supplementary Text); UACR in a casual urine sample ≥ 50 mg/gCr (≥ 5.655 mg/mmol); median UACR ≥ 50 mg/gCr and < 300 mg/gCr (≥ 5.655 mg/mmol and < 33.93 mg/mmol) in first morning void urine samples collected during 3 days at 2 weeks before randomization; and patients with a diastolic blood pressure (BP) of < 100 mmHg and systolic BP of < 160 mmHg. The main exclusion criteria of the dose–response study are summarized in the Supplementary Text.

### Endpoints

#### Primary efficacy endpoint

The primary endpoint was the percent change from baseline in UACR (first morning void urine) at 24 weeks after randomization (in the dose–response study). The exploratory endpoint was percent change from baseline in UACR according to concomitant ACE-I/ARB use.

#### Secondary efficacy endpoints

The proportion of patients achieving UACR remission (UACR of < 30 mg/gCr and a decrease in UACR of ≥ 30% from baseline) at 24 weeks (dose–response study) and 52 weeks (extension study) after randomization was evaluated as a secondary efficacy endpoint. Other secondary efficacy endpoints were percent change from baseline in UACR at 52 weeks after randomization, time-course changes in the percent change from baseline in UACR, and change from baseline in BP at 24 weeks in all patients and stratified by baseline BP and concomitant ACE-I/ARB use.

#### Safety

Safety parameters were percent change from baseline in eGFR at 52 weeks after randomization, time-course changes in the percent change from baseline in eGFR at 24 weeks and up to 52 weeks after randomization, change from baseline in serum potassium level at 52 weeks after randomization, time-course changes in the change from baseline in serum potassium level at 24 weeks and up to 52 weeks after randomization, and incidences of adverse events (AEs) and adverse drug reactions (ADRs).

### Statistical analysis

Details of the sample size calculations for the dose–response study, definitions of analysis sets (full analysis set [FAS], and safety analysis set), and descriptions of the secondary analysis of the primary efficacy endpoint are provided in the Supplementary Text. Descriptive statistics were used for baseline demographic and clinical characteristics, with *n* (%) for categorical variables and mean (standard deviation) or median (minimum and maximum) for continuous variables. For the primary and secondary endpoints, the number of patients, the geometric mean of the ratio of the UACR relative to baseline and the 95% confidence interval (CI), and the percent change from baseline were calculated. Analysis of covariance was used for intergroup comparisons. For missing efficacy data at the end of treatment, missing values were replaced using the last observation carried forward (LOCF) method. The statistical software used was SAS version 9.4 (SAS Institute, Cary, NC, USA).

## Results

### Participants

Of the 791 patients enrolled in the dose–response study, 498 patients dropped out of the study during the run-in period (most commonly because the UACR inclusion criterion was unmet), and 293 patients were randomly assigned to treatment (73 and 73, 74, and 73 patients, respectively, to the placebo and apararenone 2.5 mg, 5 mg, and 10 mg groups) (Supplementary Figure 2). Thirty-two patients discontinued; the most common reason was AEs in 12 patients. In total, 261 patients completed the dose–response treatment period. Of 241 patients enrolled in the extension phase, we focused on 188 patients in Group 1 (62, 64, and 62 patients in the apararenone 2.5, 5, and 10 mg groups, respectively).

At baseline, in the FAS, 75.7% of patients were male with a mean age of 61.8 years. In the dose–response study, over 60% of patients in each group were concomitantly using ACE-I/ARB (Table 1). No marked differences were noted in patient demographic and clinical characteristics between the dose–response study and extension study (Supplementary Table 1).

**Table 1:** Baseline demographic and clinical characteristics of patients in the dose–response study (full analysis set)

	Placebo	Apararenone 2.5 mg	Apararenone 5 mg	Apararenone 10 mg
N	72	73	74	73
Sex/male, *n* (%)	54 (75.0)	52 (71.2)	57 (77.0)	58 (79.5)
Age, years	60.1 (10.0)	63.2 (8.5)	61.7 (9.0)	62.1 (9.5)
60–69, *n* (%)	34 (47.2)	33 (45.2)	33 (44.6)	34 (46.6)
70–75, *n* (%)	10 (13.9)	18 (24.7)	15 (20.3)	15 (20.5)
BMI (kg/m^2^)	27.00 (4.64)	26.13 (3.91)	26.96 (4.94)	27.01 (4.76)
Body weight (kg)	73.97 (17.84)	68.97 (13.06)	73.39 (15.02)	73.88 (17.29)
Duration of T2DM, years	12.82 (7.61)	13.95 (10.16)	14.64 (9.71)	14.54 (8.06)
Use of ACE-I/ARB, yes, *n* (%)	46 (63.9)	47 (64.4)	47 (63.5)	47 (64.4)
UACR (mg/gCr)	141.62 (88.23)	151.18 (88.37)	131.91 (87.54)	130.20 (68.34)
Median (range)	116.25 (42.0–472.6)	132.10 (26.0–478.2)	111.20 (33.0–451.0)	108.90 (42.7–332.9)
eGFR (mL/min/1.73 m^2^)	77.5 (20.5)	70.9 (17.9)	78.3 (20.4)	73.0 (21.8)
HbA1c [NGSP], %	7.08 (0.80)	7.02 (0.86)	7.32 (1.01)	7.28 (0.92)
SBP (mmHg)	133.4 (11.8)	136.6 (11.8)	136.0 (11.6)	135.0 (12.5)
DBP (mmHg)	77.7 (9.1)	77.7 (9.4)	77.0 (10.3)	79.0 (10.1)
Serum potassium (mmol/L)	4.24 (0.31)	4.27 (0.28)	4.28 (0.26)	4.29 (0.29)

Data in the table are mean (SD), unless otherwise indicated

*BMI* body mass index, *T2DM* type 2 diabetes mellitus, *ACE-I* angiotensin converting enzyme inhibitor, *ARB* angiotensin II receptor antagonist, *UACR* urine albumin-to-creatinine ratio, *eGFR* estimated glomerular filtration rate, *HbA1c* glycated hemoglobin, *NGSP* National Glycohemoglobin Standardization Program, *SBP* systolic blood pressure, *DBP* diastolic blood pressure, *SD* standard deviation

### Endpoints

#### Primary efficacy endpoint

**Fig. 1:**
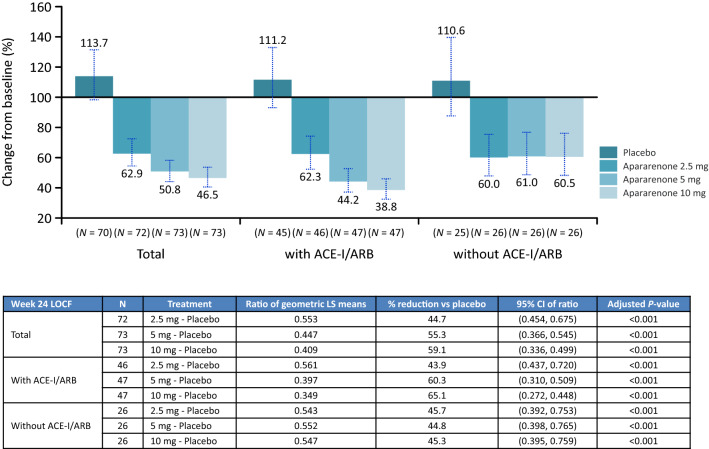
Percent change from baseline in UACR at 24 weeks after randomization (in all patients and stratified by concomitant ACE-I/ARB use). *ACE-I* angiotensin-converting enzyme inhibitors, *ARB* angiotensin II receptor blocker, *CI* confidence interval, *LOCF* last observation carried forward, *LS* least squares, *UACR* urine albumin to creatine ratio

From baseline to week 24, UACR (first morning void urine) (95% CI), as a percentage of the baseline level (= 100%), decreased significantly in all apararenone groups (2.5 mg, 62.9% [54.6–72.5]; 5 mg, 50.8% [44.1–58.4]; and 10 mg, 46.5% [40.4–53.5]) but not the placebo group (113.7% [98.5–131.2]) (*P* < 0.001 for all apararenone groups versus placebo), in a dose-dependent manner, and regardless of concomitant ACE-I/ARB use (Fig. 1). UACR decreased in patients with and without concomitant ACE-I/ARB use. Decreases in UACR up to 24 weeks after randomization (LOCF) were significant for apararenone 2.5 mg, 5 mg, and 10 mg groups compared with placebo (*P* < 0.001, all) (Supplementary Figure 3).

#### Secondary efficacy endpoints

**Fig. 2:**
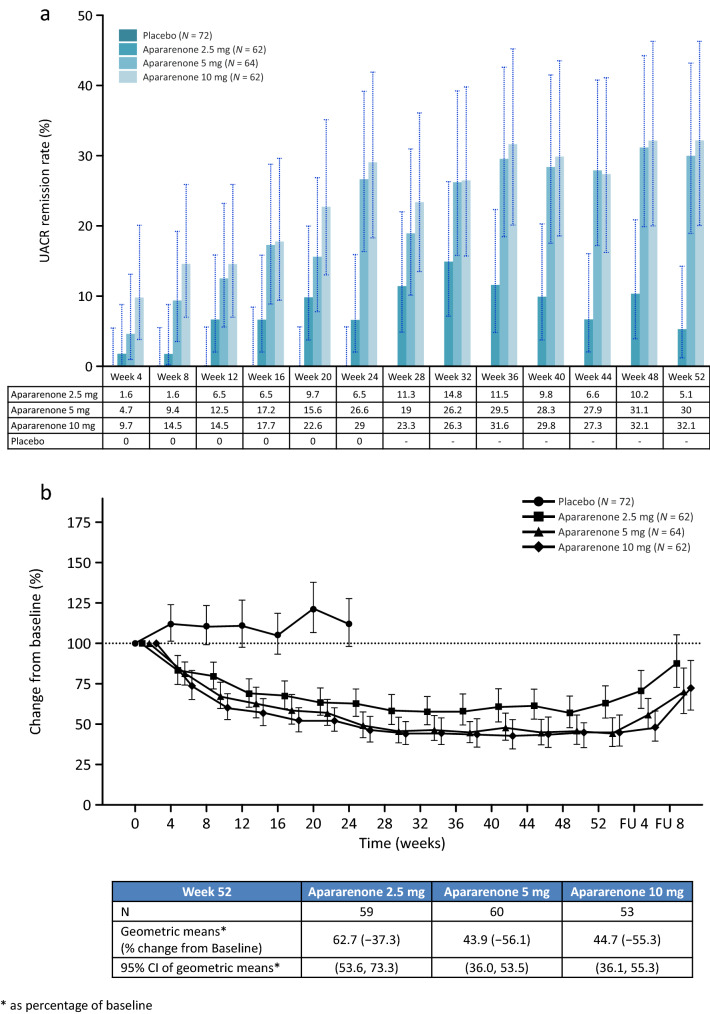
**a** UACR remission rate at each time point up to 52 weeks after randomization. *UACR* urine albumin to creatine ratio. Placebo group data from the 24-week dose–response study only. Apararenone treatment data from the 24-week dose–response study and the 28-week extension study. **b** Time-course of percent changes from baseline in UACR up to 52 weeks after randomization. *CI* confidence interval, *FU* follow-up, *UACR* urine albumin to creatine ratio. Geometric LS mean (95% CI). Placebo group data from the 24-week dose–response study only. Apararenone treatment data from the 24-week dose–response study and the 28-week extension study

At 24 weeks, the UACR remission rates (95% CI) were 0.0% (0.0–5.6), 7.8% (2.6–17.3), 29.0% (18.7–41.2), and 28.1% (17.6–40.8) in the placebo group and apararenone 2.5 mg, 5 mg, and 10 mg groups, respectively (Supplementary Table 2). The proportion of patients achieving UACR remission was higher in patients concomitantly receiving ACE-I/ARB than those not receiving ACE-I/ARB. The UACR remission was maintained up to week 52 (Fig. 2a). The percent change from baseline in UACR at 52 weeks after randomization was − 37.3%, − 56.1%, and − 55.3% in the apararenone 2.5 mg, 5 mg, and 10 mg groups, respectively (Fig. 2b).

**Fig. 3:**
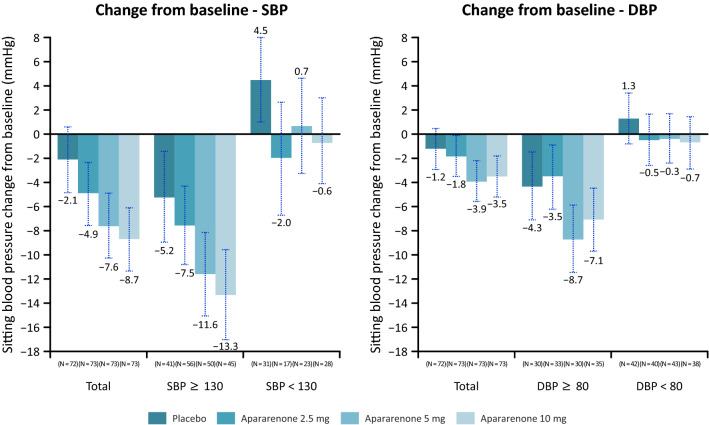
Change in blood pressure at 24 weeks after randomization (in all patients and stratified by baseline SBP and DBP). Mean (standard deviation). *DBP* diastolic blood pressure, *SBP* systolic blood pressure

Regarding changes from baseline in BP at week 24, no marked antihypertensive effect was observed in the group with normal baseline BP (Fig. 3). The decrease in BP was larger in patients who concomitantly received ACE-I/ARB (Supplementary Figure 4).

#### Safety

**Fig. 4:**
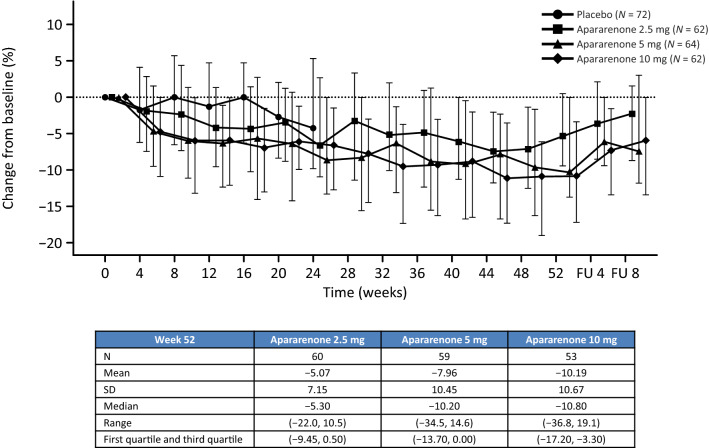
Time-course of percent changes from baseline in eGFR up to 52 weeks after randomization. *eGFR* estimated glomerular filtration rate, *FU* follow-up, *SD* standard deviation, *Q1* first quartile, *Q3* third quartile. Median (Q1/Q3). Placebo group data from the 24-week dose–response study only. Apararenone treatment data from the 24-week dose–response study and the 28-week extension study

The percent change (median [range]) from baseline in eGFR at 24 weeks after randomization (dose–response study) was − 6.75% (− 23.7, 10.3), − 8.80% (− 53.7, 23.5), and − 6.60% (− 31.5, 10.8) in the apararenone 2.5 mg, 5 mg group, and 10 mg groups compared with − 4.25% in the placebo group (Supplementary Figure 5). The percent change (median [range]) from baseline in eGFR at 52 weeks after randomization was − 5.30% (− 22.0, 10.5), − 10.20% (− 34.5, 14.6), and − 10.80% (− 36.8, 19.1) in the apararenone 2.5 mg, 5 mg, and 10 mg groups, respectively (Fig. 4). In patients showing an eGFR decrease of ≥ 30%, no ADRs associated with renal function were observed.

**Fig. 5:**
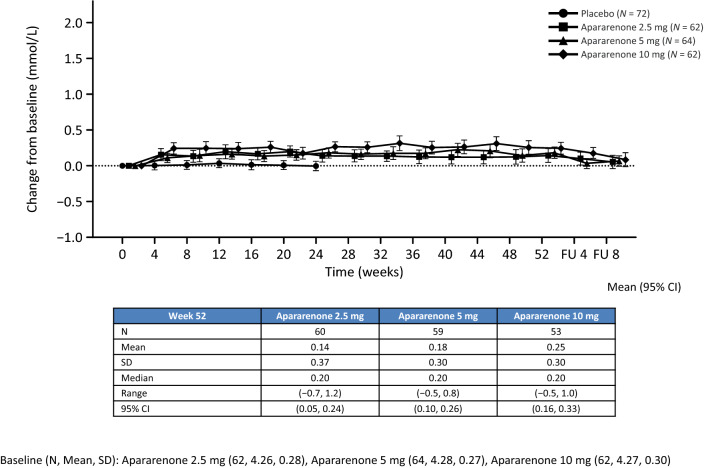
Time-course of changes from baseline in serum potassium level (measured at a central laboratory) up to 52 weeks after randomization. Mean (95% CI). *CI* confidence interval, *FU* follow-up, *SD* standard deviation. Placebo group data from the 24-week dose–response study only. Apararenone treatment data from the 24-week dose–response study and the 28-week extension study

From baseline to 24 weeks of treatment (dose–response study), serum potassium levels began to increase from week 4 up to week 12 in the apararenone 2.5 mg and 5 mg groups (median 0.10 mmol/L; both), at which point the serum potassium levels remained stable. In the apararenone 10 mg group, serum potassium levels increased to a median of 0.30 mmol/L at week 4 and remained stable up to week 16, at which point it started to decline (Supplementary Figure 6). Mean (95% CI) changes from baseline at week 52 in serum potassium level in the apararenone 2.5 mg, 5 mg, and 10 mg groups were 0.14 mmol/L (0.06, 0.22), 0.18 mmol/L (0.1, 0.26), and 0.25 (0.16, 0.33) mmol/L, respectively (Fig. 5).

In the extension study, 72.6%, 82.8%, and 93.5% of patients in the apararenone 2.5 mg, 5 mg, and 10 mg groups, respectively, presented an AE. No deaths occurred during the study (Supplementary Table 3). The most common AEs (with a frequency of ≥ 4% [by preferred term]) in any treatment group (extension study) are shown in Supplementary Table 4. ADRs occurred in three patients (4.8%), eight patients (12.5%), and three patients (4.8%) in the apararenone 2.5 mg, 5 mg, and 10 mg groups, respectively (Table 2). Reasons for discontinuation are shown in Supplementary Table 5 and Supplementary Table 6.

**Table 2:** ADRs and ADRs reported in ≥ 2  patients in any treatment group (extension study)

	Apararenone 2.5 mg *N* = 62	Apararenone 5 mg *N* = 64	Apararenone 10 mg *N* = 62
ADRs	3 (4.8)	8 (12.5)	3 (4.8)
Death	0	0	0
Serious ADRs	0	0	0
Patients who discontinued treatment because of an ADR	0	2 (3.1)	1 (1.6)
Hyperkalemia	0	0	0
Blood potassium increased	0	0	1 (1.6)
ADRs reported in ≥ 2 patients in any treatment group			
Vascular disorders	0	3 (4.7)	0
Hypotension	0	2 (3.1)	0
Investigations	0	3 (4.7)	0
Blood potassium increased	0	2 (3.1)	0

Data are reported as *n* (%)

*ADR* adverse drug reaction

## Discussion

To our knowledge, this is the first phase 2 dose–response and open-label extension phase study of apararenone in patients with early-stage DN. The UACR-lowering effect of apararenone orally administered at 2.5–10 mg/day for 24 weeks in patients with stage 2 DN was confirmed to be statistically significant (*P* < 0.001; all) compared with placebo. Both 5 mg/day and 10 mg/day apararenone groups had a greater reduction in UACR than the 2.5 mg/day apararenone group. While none of the patients receiving placebo achieved remission, the remission rates increased with the dose in the apararenone groups; the highest remission rates were achieved in the apararenone 5 mg (29.0%) and 10 mg (28.1%) groups. After 24 weeks, the percent reduction from baseline in the UACR was around 40% in the apararenone 2.5 mg group and around 50% in both the apararenone 5 mg and 10 mg groups. After 52 weeks, the percent reduction from baseline in the UACR was around 60% in both the apararenone 5 mg and 10 mg groups. In each treatment group, the decrease in the UACR started at 4 weeks after randomization, was confirmed at 24 weeks, and persisted until 52 weeks after randomization.

A decrease in BP from baseline to week 24 was observed with each apararenone dose. Notably, patients with higher baseline BP presented an anti-hypertensive effect, while BP was unaffected among patients whose baseline BP was normal. Further, the decrease in BP seemed marked among patients who were concomitantly taking ACE-I/ARB. Clinical studies on MRAs (i.e., spironolactone and eplerenone) in patients with DN showed that these drugs inhibit urine albumin excretion [14, 15]. The present findings seem to further validate the efficacy of MRAs for DN. Such effects could be attributable to improvement in renal hemodynamics (i.e., in excessive filtration) and correction of excessive sodium accumulation in the body [16, 17], but further studies are needed.

Recent meta-analyses have reported that a decrease of ≥ 30% in the UACR can reduce the event risk [18, 19]. Therefore, the UACR-lowering effect of apararenone could also be significant from the clinical viewpoint. In the present study, apararenone lowered UACR in patients with and without concomitant ACE-I/ARB use. Additionally, the UACR-lowering effect and proportion of patients achieving UACR remission were greater in patients with concomitant ACE-I/ARB use compared with those not using ACE-I/ARB. These findings suggest that apararenone would be useful in lowering UACR in patients with DN who are at risk of presenting increased serum potassium levels, which would cause difficulties when using ACE-I/ARB. Further, the finding that a marked antihypertensive effect was only shown in the group with high baseline BP suggests that apararenone may produce an antihypertensive effect according to the pathological condition of DN. The antihypertensive effect was also greater among patients concomitantly taking ACE-I/ARB. MRAs could result in sustained improvement of fibrosis of various organs throughout the body [20–24], and our findings suggest that apararenone could improve DN. Thus, apararenone would be expected to improve fibrotic disease in the liver and other organs.

During 52 weeks of treatment with apararenone, eGFR seemed to slowly decrease by week 12 up to week 52 of treatment in each group. It was not possible to confirm any clear achievement of steady-state eGFR within the 52-week treatment. Notably, eGFR tended to return to baseline in all treatment groups at the end of treatment suggesting that the effects of apararenone on renal function did not result in organic changes or damage. While concomitant ACE-I/ARB use seemed to be associated with a marked decrease in eGFR, patients showing an eGFR decrease of ≥ 30% did not present ADRs associated with renal function. These results also suggest that these effects, including the UACR-lowering effect, are at least partially reversible.

In the present study, patients with a potassium level of ≥ 6.0 mmol/L at two consecutive measurements on the same day during the study period were required to discontinue treatment because the increase in serum potassium was considered to be a risk specific to the drug. Nonetheless, the mean change in potassium levels from baseline was around 0.2–0.3 mmol/L in patients who continued the oral 52-week treatment with apararenone. Additionally, after roughly 4 weeks of treatment, potassium levels became stable and no tendency for continuous increase in potassium levels was observed during long-term treatment. Thus, no safety concerns related to serum potassium level increases were raised.

While the AE incidence tended to increase with increasing dose, the ADR incidence did not increase with the dose. No ADRs occurred in two or more patients in any of the treatment groups. Reported AEs were generally controlled through regular monitoring and no deaths were reported.

Widespread use of classic MRAs, which have poor specificity for MRs, is limited [21]. For instance, spironolactone is associated with ADRs such as gynecomastia, breast tenderness, and menstrual irregularity [14]. Eplerenone binds to androgen and progesterone receptors with weaker affinity than spironolactone but is contraindicated due to the increased risk of hyperkalemia in patients with moderate or severe renal dysfunction and diabetic patients with albuminuria or proteinuria [15]. Apararenone is a non-steroidal compound that has highly selective MRA activity, and thus, is not associated with such ADRs. Though head-to-head comparisons are needed, we expect that the safety profile of apararenone will be at least equivalent to that of other drugs.

This study had several limitations. The sample sizes of both the dose–response and extension studies were relatively small at 293 patients and 241 patients, respectively. In the evaluation of eGFR, only data up to week 24 were available from the placebo group as a comparator.

## Conclusion

The UACR-lowering effect of apararenone orally administered at doses of 2.5 mg, 5 mg, and 10 mg once daily for 24 weeks in patients with stage 2 DN was confirmed with or without concomitant ACE-I/ARB use. The present results showed the safety and tolerability of apararenone at 2.5–10 mg/day for up to 52 weeks, and suggest that the most appropriate clinical doses of apararenone are 5 mg and 10 mg once daily.

## Electronic supplementary material

Below is the link to the electronic supplementary material.

Supplementary file1 (DOCX 61 kb)

Supplementary file2 (TIF 324 kb)

Supplementary file3 (TIF 310 kb)

Supplementary file4 (TIF 312 kb)

Supplementary file5 (TIF 340 kb)

Supplementary file6 (TIF 290 kb)

Supplementary file7 (TIF 282 kb)

## Data Availability

The data that support the findings of this study are available from the corresponding author, upon reasonable request.
