# Supplementary material for: Apararenone in patients with diabetic nephropathy: results of a randomized, double-blind, placebo-controlled phase 2 dose–response study and open-label extension study
Source: Clin Exp Nephrol. 2020 Sep 24;25(2):120–30. doi: 10.1007/s10157-020-01963-z (PMC7880964; doi:10.1007/s10157-020-01963-z)
Supplement: Supplementary file 1 — Supplementary file1 (DOCX 61 kb) [file 10157_2020_1963_MOESM1_ESM.docx]

**SUPPLEMENTARY MATERIALS**

**Supplementary Text**

List of investigators

Endpoints

Prohibited concomitant medications

Main exclusion criteria

Randomization and blinding

Treatment

Estimated glomerular filtration rate calculation

Statistical Methods

*Sample size calculations for the dose–response study*

*Definitions of analytical populations*

*Secondary analysis of the primary efficacy measure*

**Fig. S1** Overall study design (dose–response and extension studies)

*ACE-I* angiotensin converting enzyme inhibitor, *ARB* angiotensin II receptor antagonist, *UACR* urine albumin-to-creatinine ratio

**Fig. S2** Overall patient disposition (dose–response and extension studies).

*pts,* patients

**Fig. S3** Time-course of percent changes from baseline in UACR up to 24 weeks after randomization

*CI* confidence interval, *LOCF* last observation carried forward, *LS* least squares, *UACR* urine albumin-to-creatinine ratio

**Fig. S4** Change in blood pressure at 24 weeks (LOCF) after randomization (in all patients and stratified by concomitant ACE-I/ARB use)

*ACE-I* angiotensin converting enzyme inhibitor, *ARB* angiotensin II receptor antagonist

**Fig. S5** Time-course of percent changes from baseline in eGFR up to 24 weeks after randomization

*CI* confidence interval, *eGFR* estimated glomerular filtration rate, *SD* standard deviation, *Q1* first quartile, *Q3* third quartile

**Fig. S6** Time-course of changes from baseline in serum potassium level (measured at a central laboratory) up to 24 weeks after randomization

*CI* confidence interval, *SD* standard deviation

**Supplementary Text**

**List of Investigators**

A total of 49 study sites participated in the dose–response study and in the extension study. Investigators included: Izumi Tsunematsu, Touei Hospital; Michio Eguchi, Ishikari Hospital; Yuri Ono, Yuri Ono Clinic; Yuichi Nakamura, Nakamura Digestive Organ Internal Medicine Clinic; Kenichi Imamura, Imamura Clinic; Tsukiko Tomiyama, Naika Ohisama Clinic; Fuminobu Okuguchi, OKUGUCHI clinic of internal medicine; Masayuki Noritake, Noritake Clinic; Takeshi Osonoi, Nakakinen Clinic; Mihoko Matsumura, Kamitsuga General Hospital; Wataru Araki, SUBARU Health Insurance Society Ota Memorial Hospital; Takashi Nagai, Public Tomioka General Hospital; Toshio Kawada, Kawada clinic; Takatoshi Otani, Ota Diabetes Clinic; (dose–response study)Toshiko Narahashi, (extension study)Yuichiro Makita, Koshigaya Municipal Hospital; Shunichiro Onishi, Asahi General Hospital; Yusuke Fujino, New Tokyo Heart Clinic; Takahiko Tokuyama, THY Tokuyama Clinic; Toru Hiyoshi, Japanese Red Cross Medical Center; Kazuyuki Mizuyama, Doujin Memorial Medical Foundation Meiwa Hospital; Takanobu Itoi, Japan Community Healthcare Organization Tokyo Takanawa Hospital; Kageki Ito, Ito Clinic; Mitsutoshi Kato, Kato clinic of internal medicine; Koki Shin, Shin Clinic; Tatsushi Sugiura, SEIWA CLINIC; Munechika Noguchi, Medical Corporation IHL Shinagawa East One Medical Clinic; Osamu Tomonaga, Tomonaga Clinic; Kazuo Aihara, Aihara naika clinic; Kazuo Kanno, Medical Corporation Ouitsukai Kanno Naika; Arihiro Kiyosue, Tokyo-Eki Center-building Clinic; Yoshihiko Suzuki, HDC ATLAS CLINIC; Hideki Nishimura, Kumanomae Nishimura Medical Clinic; Daisuke Suzuki, STOP DM SUZUKI DIABETES CLINIC; Kiyoshi Izumino, Fujikoshi Hospital; Hajime Fujita, Fujita Internal Medicine Clinic; Michio Nakagawa, Matsumoto Nakagawa Hospital; (dose–response study)Masanori Yoshida, (extension study)Norio Takahashi, Nagoya Ekisaikai Hospital; Nobuyasu Noritake, Daiyukai Health System Daiyukai Clinic; Noriaki Utsu, Social Corporation Keigakukai Minamiosaka Hospital; Haruhiko Kouhara, National Hospital Organization Osaka Minami Medical Center; Yorihiko Higashino, Medical Corporation Aishinkai Higashi Takarazuka Satoh Hospital; Yasuhiro Ono, Takagi Hospital; Shinichi Tanaka, Shin Yukuhashi Hospital; Masao Ishii, Fukuoka Wajiro Hospital; Shigeru Fujii, Fukuoka Shinmizumaki Hospital; Masayuki Kaneko, Medical Corporation Keiwakai, Oita Oka Hospital; Takamoto Kodera, SAIKI CENTRAL HOSPITAL; Yoshito Inobe, Medical Corporation Ikeikai Inobe Funai Clinic; Shuji Nakamura, Heiwadai Hospital

**Objective of the extension study**

The objective of the extension study was to confirm the long-term safety of apararenone in patients with early-stage diabetic nephropathy.

**Endpoints**

To calculate the primary endpoint, percent change from baseline in urine albumin to creatine ratio (UACR) (first morning void urine) at 24 weeks after randomization (in the dose–response study), the median value of three measurements taken over 3 days in the first morning void urine at each assessment time point was used for the UACR values. No primary efficacy endpoint was specified for the extension study because the later was conducted as an extension of the dose–response study to investigate the efficacy and safety of apararenone with long-term use. UACR remission rate at 24 weeks was also evaluated according to concomitant angiotensin-converting enzyme inhibitors (ACE-I) and angiotensin II receptor blockers (ARBs) use.

Serum potassium levels were measured at a central laboratory and patients with potassium levels of ≥6.0 mmol/L at two consecutive measurements on the same day during the study period were required to discontinue treatment. Adverse events were classified by MedDRA preferred term and system organ class and by severity.

**Prohibited concomitant medications**

The prohibited concomitant treatments in the dose–response and extension studies were potassium-sparing diuretics, including mineralocorticoid receptor antagonists and combination drugs (i.e., amiloride, triamterene, spironolactone, eplerenone, and potassium canrenoate); renin inhibitors; potassium supplements; calcineurin inhibitors (cyclosporine, tacrolimus, and pimecrolimus); heparin, ketoconazole (only oral formulations), trimethoprim, and pentamidine; celecoxib, warfarin, phenytoin; alfentanil, astemizole, cisapride, dihydroergotamine, ergotamine, fentanyl, pimozide, quinidine, sirolimus, and terfenadine; phosphate binders (applicable only to sevelamer hydrochloride and bixalomer), potassium binders, and anion exchange resins; and spherical adsorbing carbon.

**Criteria for use of an ACE-I or ARB**

Patients receiving treatment with an ACE-I or ARB must have received the treatment for at least 12 weeks before the start of the run-in period. The start of new treatment with an ACE-I or ARB was prohibited from 4 weeks before the start of the run-in period to the last observation during the follow-up period. The combined use of ACE-I and ARB was to be avoided and the dosage regimen was to remain constant from 4 weeks before the start of the run-in period to the last observation during the follow-up period. Patients who had not received treatment with an ACE-I or ARB were prohibited from using an ACE-I or ARB from at least 12 weeks before the start of the run-in period to the last observation during the follow-up period.

**Main exclusion criteria**

The main exclusion criteria of the dose–response study were type 1 diabetes mellitus, diabetes mellitus resulting from pancreatic disorder, or secondary forms of diabetes (e.g., Cushing’s syndrome, steroid-induced diabetes); non-diabetic kidney disease; history of nephrectomy, renal transplant, or dialysis treatment; central laboratory serum potassium level that met any of the following criteria before randomization: estimated glomerular filtration rate (eGFR) of 30 to 59 mL/min/ 1.73 m^2^ and serum potassium level <3.5 or >4.7 mmol/L or eGFR of ≥60 mL/min/ 1.73 m^2^ and serum potassium level <3.5 or >5.0 mmol/L; clinically significant or symptomatic hypotension; long QT syndrome or Torsades de Pointes; heart failure New York Heart Association Class III-IV; body mass index of >45 kg/m^2^; stroke, acute limb ischemia, myocardial infarction; level of aspartate aminotransferase or alanine aminotransferase ≥3 times the upper limit of normal; patients with clinically significant thyroid gland disorders, evidence of acute ischemia on 12-lead electrocardiogram; history of malignancy 5 years earlier; active urinary tract infection; acute kidney injury; serious concomitant medical disease; history of alcohol abuse or drug abuse; use of prohibited concomitant medications; women who were pregnant, nursing, or possibly pregnant; or ineligibility for the study by the investigator.

Patients who progressed to overt nephropathy (worsened) in the dose-response study were excluded from the extension study if they presented a median first morning void urine UACR (value of urine collected for 3 days) ≥300 mg/gCr and presented an increase of ≥30% from baseline in UACR at 20 weeks after randomization in the dose–response study, or met any of the main exclusion criteria.

**Randomization and blinding**

A computer-generated randomization was conducted using a permuted block method based on the presence or absence of concomitant ACE-I or ARB and UACR obtained two weeks prior. At 20 weeks after randomization, patients from the dose–response study who received placebo were randomly assigned to apararenone 2.5 mg, 5 mg, or 10 mg using a permuted block method. Patients from the dose–response study who received apararenone continued apararenone treatment at the same dose assigned in the dose–response study.

All tablets were identical in appearance, and the packaging and labeling were designed to mask the participant, care provider, investigator, and outcomes assessor. Double-blinding from the dose–response study was maintained until the data for all the test and observation parameters for all of the patients in the dose–response study were locked.

**Estimated glomerular filtration rate calculation**

The formula used to calculate the estimated glomerular filtration rate (eGFR) of men was eGFRcreat (mL/min/1.73 m^2^) = 194 × Cr^−1.094^ × age^−0.287^. The formula used to calculate the eGFR of women was eGFRcreat (mL/min/1.73 m^2^) = 194 × Cr^−1.094^ × age^−0.287^ × 0.739.

**Statistical Methods**

***Sample size calculations for the dose–response study***

Based on previous apararenone studies (data on file), the target sample size was investigated by simulation using the step-down Dunnett test considering multiplicity. Supposing that the dose–response curve of the percentage decrease in urine albumin to creatine ratio ([1 − geometric mean] × 100) was placebo <2.5 mg = 5 mg = 10 mg, and under the estimation that the percentage decrease was 40% in the apararenone group and 10% in the placebo group and that the common standard deviation of the log-transformed value was 0.6, the sample size required to demonstrate statistically significant efficacy over placebo at all apararenone doses (power, 90%) was calculated (62 patients) and considering the number of dropouts, a target sample size of 70 patients per group was determined.

***Definitions of analytical populations***

The full analysis set (FAS) consisted of all randomized patients, except those without type 2 DN, those who did not take the investigational product, or those for whom no post-randomization efficacy data were available. The safety analysis set consisted of all randomized patients except those who did not take the investigational product at all or for which no post-randomization safety data were available.

***Secondary analysis of the primary efficacy measure***

In order to examine the efficacy in the 2.5 mg group, 5 mg group, and the 10 mg group over the placebo group, repeated measures analysis of variance were performed for the log-transformed value of UACR (first-morning void urine) using the change at each measurement point as the objective variable, treatment group, period, effect of interaction between treatment group and period, the presence or absence of concomitant use of ACE-I/ARB at the start of treatment as the factors, and the log-transformed value of UACR on the first day of the treatment period as the covariate. The intergroup comparisons were performed at week 24, and the multiplicity adjustment for the intergroup comparisons at week 24 was performed using the step-down Dunnett test, with the placebo group as the control. In addition, the least squares mean and 95% CIs were calculated and inverse transformed for the intergroup difference (active drug group − placebo group).

**Supplementary Table 1.** Baseline demographic and clinical characteristics of patients in the extension study

|  | **Apararenone 2.5 mg**  ***N =*  62** | **Apararenone 5 mg**  ***N =*  64** | **Apararenone 10 mg**  ***N =*  62** |
| --- | --- | --- | --- |
| Sex/ male, *n* (%) | 43 (69.4) | 48 (75.0) | 51 (82.3) |
| Age, years | 62.9 (8.7) | 62.0 (8.9) | 61.6 (9.7) |
| 60–69, n (%) | 30 (48.4) | 28 (43.8) | 27 (43.5) |
| 70–75, n (%) | 13 (21.0) | 14 (21.9) | 13 (21.0) |
| BMI, kg/m^2^ | 26.18 (3.94) | 26.61 (4.86) | 27.08 (4.98) |
| Body weight, kg | 69.10 (13.52) | 72.08 (14.87) | 74.59 (17.99) |
| Duration of T2DM, years | 13.82 (10.64) | 15.22 (9.87) | 14.09 (7.97) |
| Use of ACE-I/ARB, yes, n (%) | 40 (64.5) | 42 (65.6) | 40 (64.5) |
| UACR, mg/gCr | 147.64 (81.34) | 133.10 (90.58) | 127.19 (66.86) |
| Median (range) | 132.95 (26.0–390.8) | 111.20 (36.8–451.0) | 110.85 (42.7–332.9) |
| eGFR, mL/min/1.73 m^2^ | 72.3 (17.9) | 77.8 (20.3) | 72.8 (22.2) |
| HbA1c [NGSP], % | 6.98 (0.85) | 7.31 (1.05) | 7.23 (0.91) |
| SBP, mmHg | 136.5 (12.2) | 136.3 (12.1) | 135.7 (12.4) |
| DBP, mmHg | 77.9 (9.4) | 76.5 (10.5) | 79.7 (10.4) |
| Serum potassium, mmol/L | 4.26 (0.28) | 4.28 (0.27) | 4.27 (0.30) |

Data in the table are mean (SD), unless otherwise indicated.

*BMI* body mass index, *T2DM* type 2 diabetes mellitus, *ACE-I* angiotensin converting enzyme inhibitor, *ARB* angiotensin II receptor antagonist, *UACR* urine albumin-to-creatinine ratio, *eGFR* estimated glomerular filtration rate, *HbA1c* glycated hemoglobin, *NGSP* National Glycohemoglobin Standardization Program, *SBP* systolic blood pressure, *DBP* diastolic blood pressure, *SD* standard deviation

**Supplementary Table 2.** UACR remission rate at 24 weeks after randomization (in all patients and stratified by concomitant ACE-I/ARB use)

| **Week 24** | **Placebo (*N =* 72)** | **Apararenone**  **2.5 mg (*N =* 73)** | **Apararenone**  **5 mg (*N =* 74)** | **Apararenone**  **10 mg (*N =* 73)** |
| --- | --- | --- | --- | --- |
| N | 64 | 64 | 69 | 64 |
| Remission, n (%) | 0 (0.0) | 5 (7.8) | 20 (29.0) | 18 (28.1) |
| 95% CI of Remission | (0.0, 5.6) | (2.6, 17.3) | (18.7, 41.2) | (17.6, 40.8) |
| **With ACE-I/ARB** |  |  |  |  |
| **Week 24** | **Placebo (*N =* 46)** | **Apararenone**  **2.5 mg (*N =* 47)** | **Apararenone**  **5 mg (*N =* 47)** | **Apararenone**  **10 mg (*N =* 47)** |
| N | 41 | 40 | 45 | 41 |
| Remission, n (%) | 0 (0.0) | 2 (5.0) | 17 (37.8) | 15 (36.6) |
| 95% CI of Remission | (0.0, 8.6) | (0.6, 16.9) | (23.8, 53.5) | (22.1, 53.1) |
| **Without ACE-I/ARB** |  |  |  |  |
| **Week 24** | **Placebo (*N =* 26)** | **Apararenone**  **2.5 mg (*N =* 26)** | **Apararenone**  **5 mg (*N =* 27)** | **Apararenone**  **10 mg (*N =* 26)** |
| N | 23 | 24 | 24 | 23 |
| Remission, n (%) | 0 (0.0) | 3 (12.5) | 3 (12.5) | 3 (13.0) |
| 95% CI of Remission | (0.0, 14.8) | (2.7, 32.4) | (2.7, 32.4) | (2.8, 33.6) |

*ACE-I* angiotensin converting enzyme inhibitor, *ARB* angiotensin II receptor antagonist, *UACR* urine albumin-to-creatinine ratio, *CI* confidence interval

**Supplementary** **Table 3.** Adverse events (extension study)

|  | **Apararenone**  **2.5 mg (*N =* 62)** | **Apararenone**  **5 mg (*N =* 64)** | **Apararenone**  **10 mg (*N =* 62)** |
| --- | --- | --- | --- |
| Adverse events | 45 (72.6) | 53 (82.8) | 58 (93.5) |
| Death | 0 | 0 | 0 |
| Serious adverse events | 0 | 2 (3.1) | 6 (9.7) |
| Patients who discontinued treatment because of an adverse event | 0 | 3 (4.7) | 3 (4.8) |

Data are reported as *n* (%).

**Supplementary Table 4.** AEs with a frequency of ≥4% (by PT) in any treatment group (extension study)

| **System Organ Class** | **Apararenone**  **2.5 mg** | **Apararenone**  **5 mg** | **Apararenone**  **10 mg** |
| --- | --- | --- | --- |
| Preferred Term (MedDRA/J) | (*N =* 62) | (*N =* 64) | (*N =* 62) |
| Infections and infestations | 29 (46.8) | 33 (51.6) | 38 (61.3) |
| Nasopharyngitis | 20 (32.3) | 23 (35.9) | 27 (43.5) |
| Gastroenteritis | 2 (3.2) | 2 (3.1) | 4 (6.5) |
| Influenza | 4 (6.5) | 1 (1.6) | 2 (3.2) |
| Bronchitis | 3 (4.8) | 3 (4.7) | 1 (1.6) |
| Periodontitis | 1 (1.6) | 3 (4.7) | 1 (1.6) |
| Blood and lymphatic system disorders | 2 (3.2) | 2 (3.1) | 6 (9.7) |
| Anemia | 0 | 0 | 5 (8.1) |
| Metabolism and nutrition disorders | 12 (19.4) | 13 (20.3) | 9 (14.5) |
| Diabetes mellitus | 7 (11.3) | 4 (6.3) | 3 (4.8) |
| Hypoglycemia | 3 (4.8) | 6 (9.4) | 2 (3.2) |
| Nervous system disorders | 6 (9.7) | 8 (12.5) | 10 (16.1) |
| Dizziness | 1 (1.6) | 0 | 3 (4.8) |
| Gastrointestinal disorders | 10 (16.1) | 9 (14.1) | 12 (19.4) |
| Constipation | 0 | 2 (3.1) | 3 (4.8) |
| Periodontal disease | 3 (4.8) | 2 (3.1) | 1 (1.6) |
| Skin and subcutaneous tissue disorders | 8 (12.9) | 8 (12.5) | 10 (16.1) |
| Eczema | 5 (8.1) | 1 (1.6) | 3 (4.8) |
| Dermatitis | 0 | 0 | 3 (4.8) |
| Musculoskeletal and connective tissue disorders | 14 (22.6) | 15 (23.4) | 8 (12.9) |
| Back pain | 6 (9.7) | 6 (9.4) | 2 (3.2) |
| Muscle spasms | 3 (4.8) | 4 (6.3) | 0 |
| Investigations | 3 (4.8) | 8 (12.5) | 5 (8.1) |
| Blood creatine phosphokinase increased | 0 | 0 | 3 (4.8) |
| Blood potassium increased | 0 | 3 (4.7) | 1 (1.6) |
| Injury, poisoning and procedural complications | 8 (12.9) | 6 (9.4) | 10 (16.1) |
| Contusion | 4 (6.5) | 0 | 4 (6.5) |

Data are reported as *n* (%).

*AE* adverse event, *MedDRA* medical dictionary for regulatory activities, *PT* preferred term

**Supplementary Table 5.** Reasons for study discontinuation in all randomized patients (dose-response study)

|  | **Placebo** | **Apararenone**  **2.5 mg** | **Apararenone**  **5 mg** | **Apararenone**  **10 mg** |
| --- | --- | --- | --- | --- |
| Primary reason for discontinuation during treatment period |  |  |  |  |
| Adverse event  Hyperkalemia  Blood potassium increased | 1 (1.4)  0  0 | 5 (6.8)  2 (2.7)  0 | 2 (2.7)  0  0 | 4 (5.5)  3 (4.1)  0 |
| Physician decision | 0 | 2 (2.7) | 0 | 0 |
| Protocol deviation | 5 (6.8) | 0 | 2 (2.7) | 3 (4.1) |
| Withdrawal by patient | 1 (1.4) | 1 (1.4) | 1 (1.4) | 2 (2.7) |
| Other | 2 (2.7) | 1 (1.4) | 0 | 0 |
| Primary reason for discontinuation during the follow-up period |  |  |  |  |
| Withdrawal by patient | 0 | 1 (1.4) | 0 | 0 |

Data are reported as *n* (%)

**Supplementary Table 6.** Reasons for study discontinuation in all randomized patients (extension study)

|  | **Apararenone**  **2.5 mg** | **Apararenone**  **5 mg** | **Apararenone**  **10 mg** |
| --- | --- | --- | --- |
| Primary reason for discontinuation during treatment period |  |  |  |
| Adverse event  Hyperkalemia  Blood potassium increased | 0  0  0 | 3 (4.7)  0  0 | 3 (4.8)  0  1 (1.6) |
| Non-compliance with study drug | 1 (1.6) | 0 | 0 |
| Protocol deviation | 0 | 0 | 1 (1.6) |
| Withdrawal by patient | 0 | 0 | 2 (3.2) |
| Other | 2 (3.2) | 1 (1.6) | 3 (4.8) |
| Primary reason for discontinuation during the follow-up period |  |  |  |
| Lost to follow-up | 1 (1.6) | 0 | 0 |

Data are reported as *n* (%)
