# Supplementary figures and images for: Apararenone in patients with diabetic nephropathy: results of a randomized, double-blind, placebo-controlled phase 2 dose–response study and open-label extension study
Source: Clin Exp Nephrol. 2020 Sep 24;25(2):120–30. doi: 10.1007/s10157-020-01963-z (PMC7880964; doi:10.1007/s10157-020-01963-z)

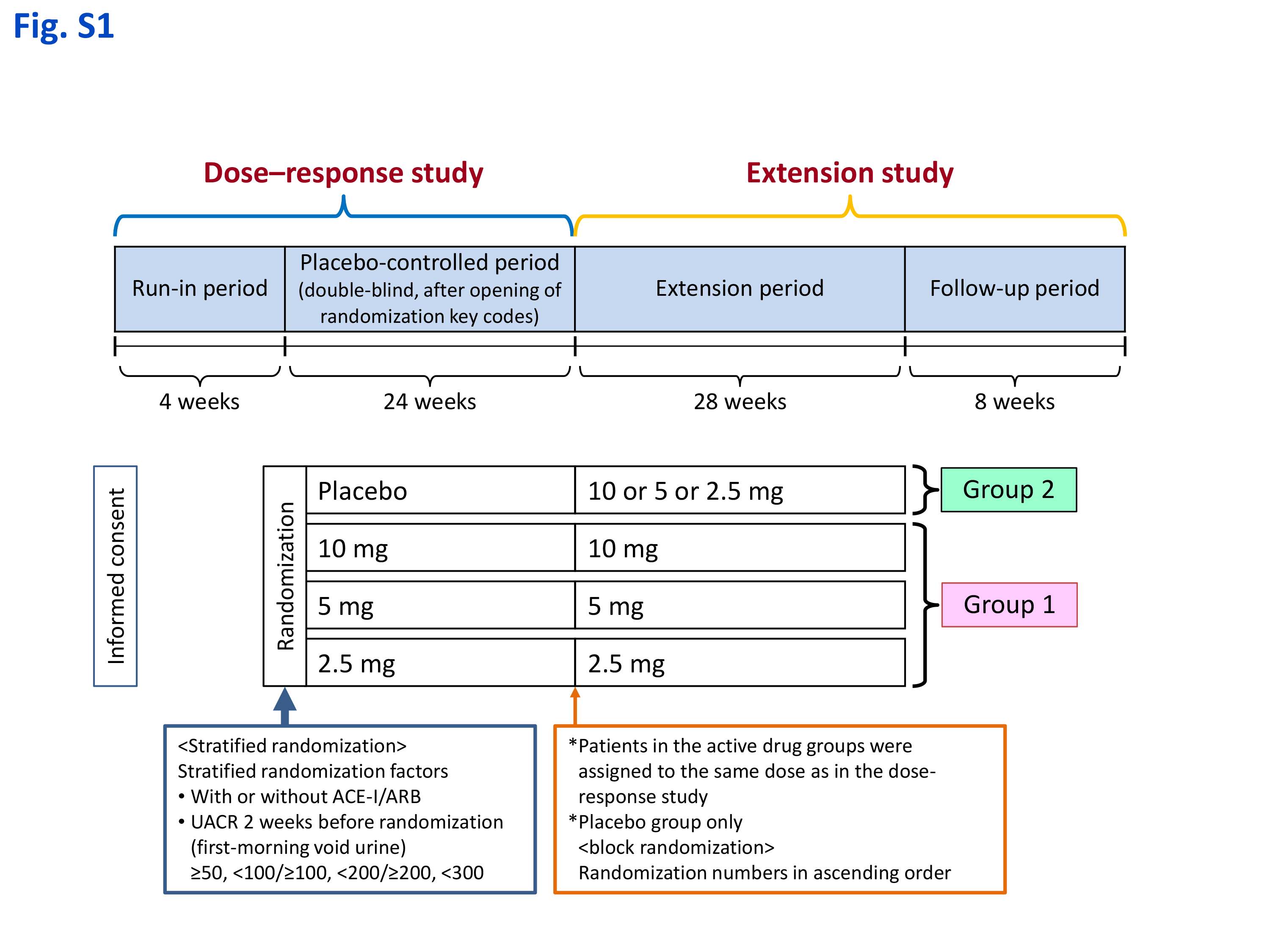

Supplement: Supplementary file 2 — Supplementary file2 (TIF 324 kb) [file 10157_2020_1963_MOESM2_ESM.tif]

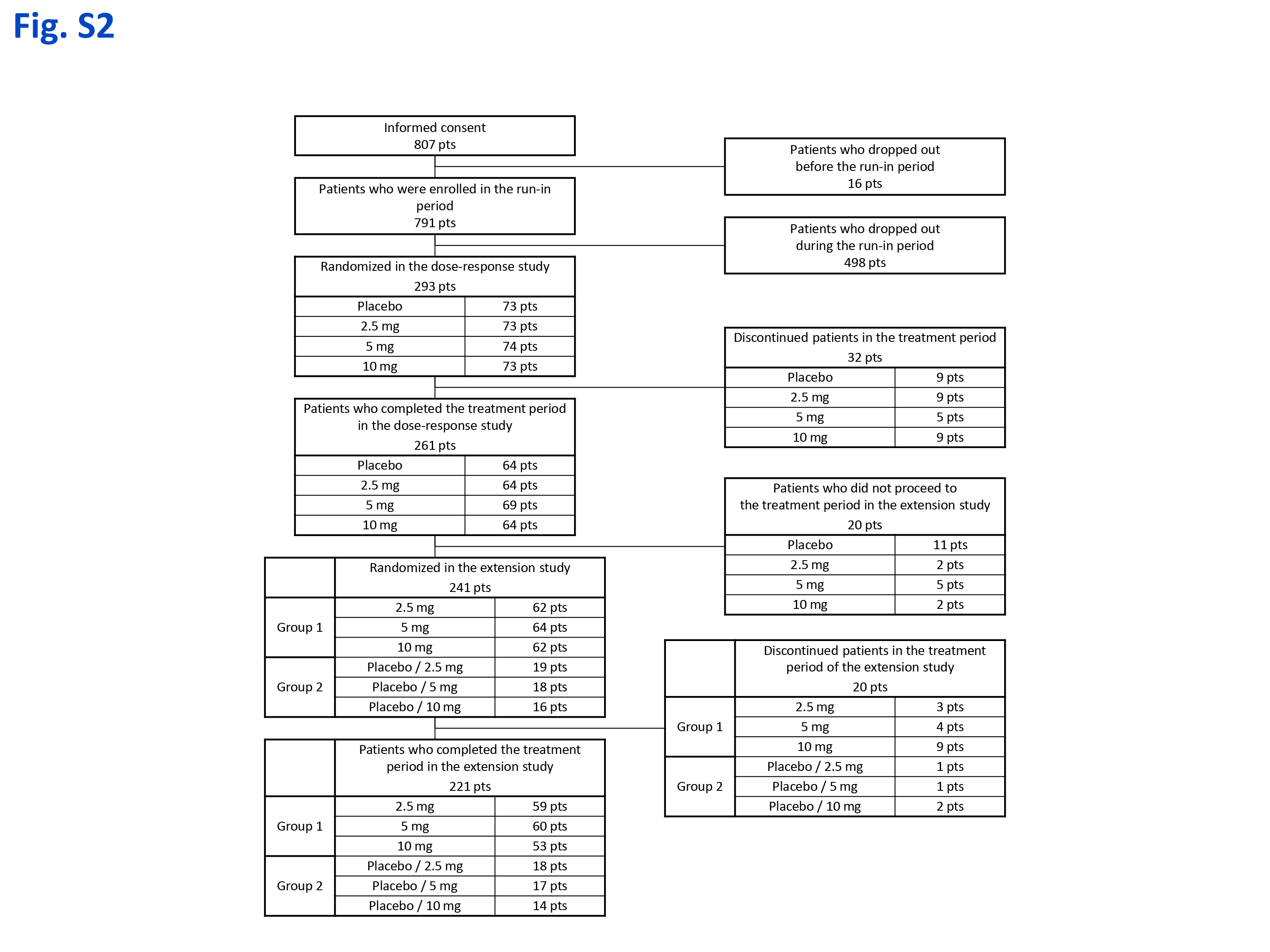

Supplement: Supplementary file 3 — Supplementary file3 (TIF 310 kb) [file 10157_2020_1963_MOESM3_ESM.tif]

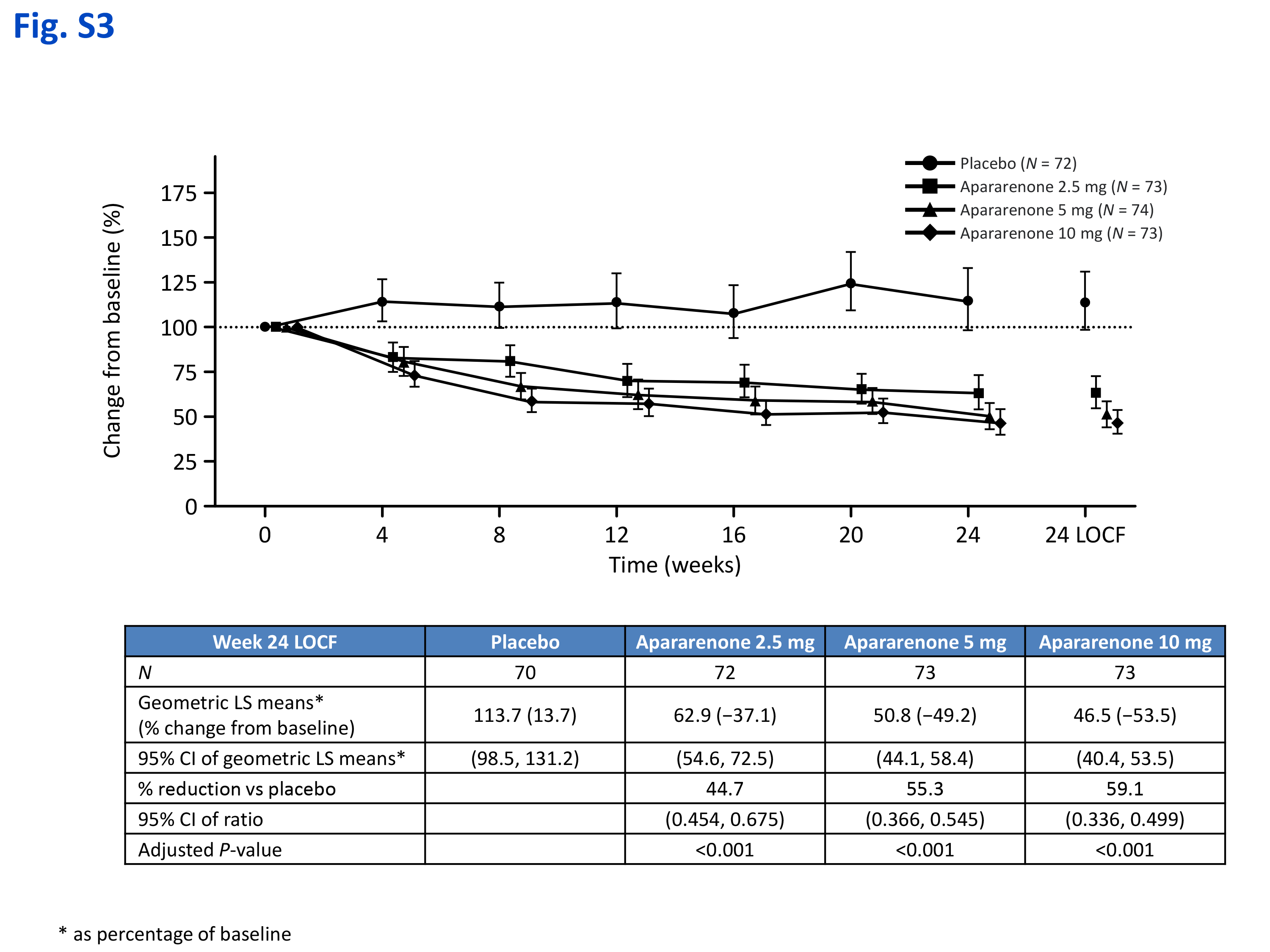

Supplement: Supplementary file 4 — Supplementary file4 (TIF 312 kb) [file 10157_2020_1963_MOESM4_ESM.tif]

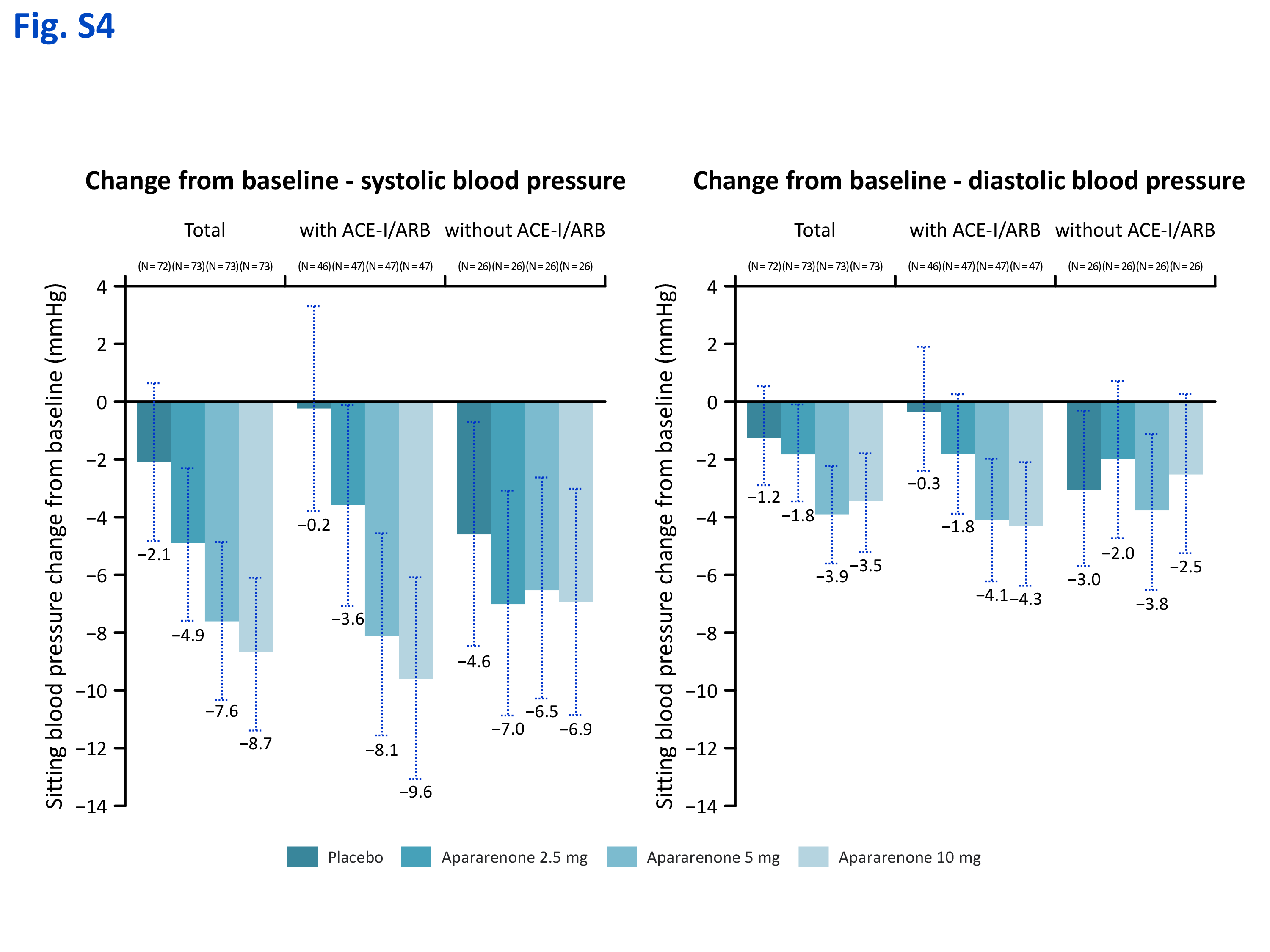

Supplement: Supplementary file 5 — Supplementary file5 (TIF 340 kb) [file 10157_2020_1963_MOESM5_ESM.tif]

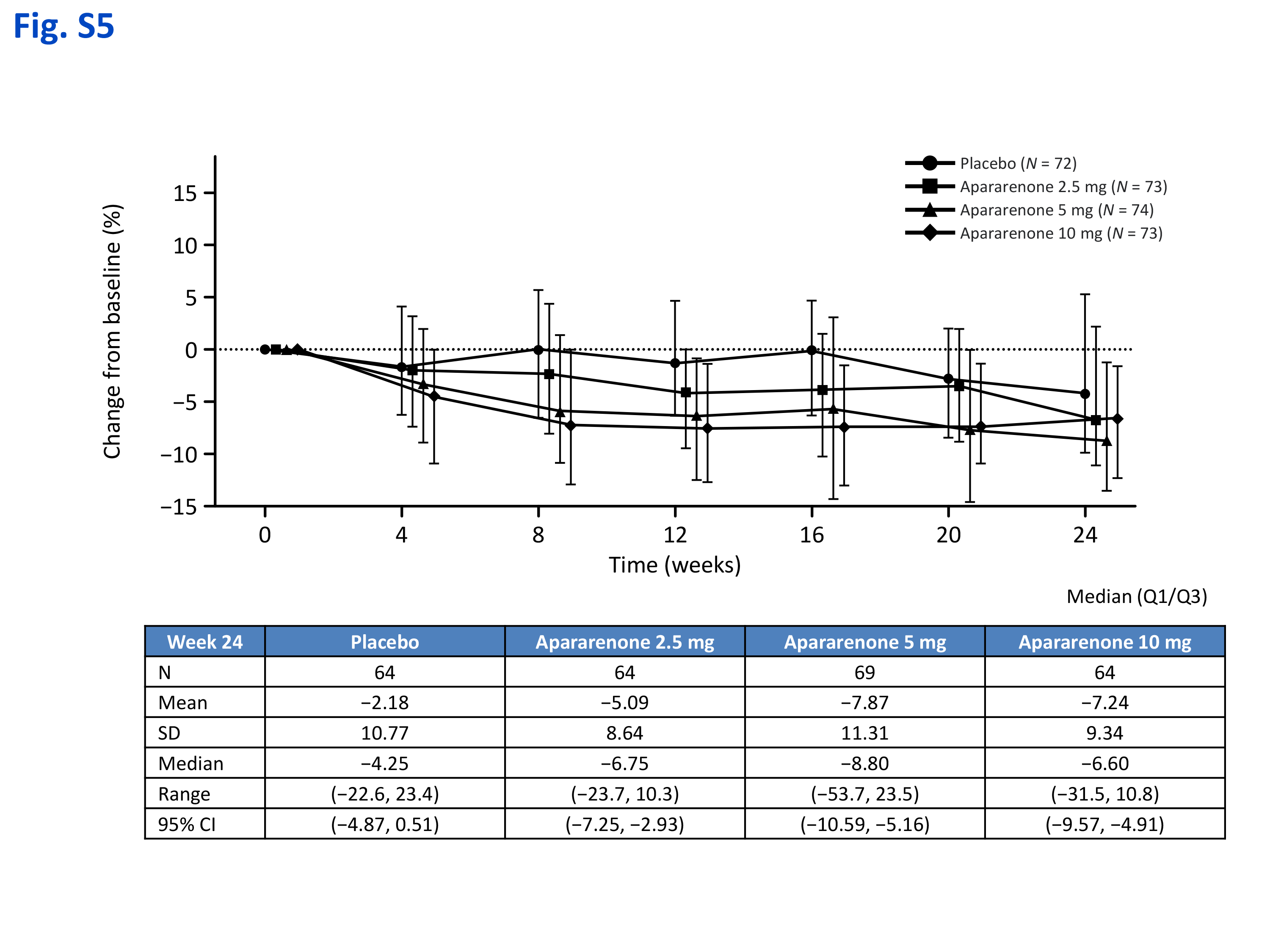

Supplement: Supplementary file 6 — Supplementary file6 (TIF 290 kb) [file 10157_2020_1963_MOESM6_ESM.tif]

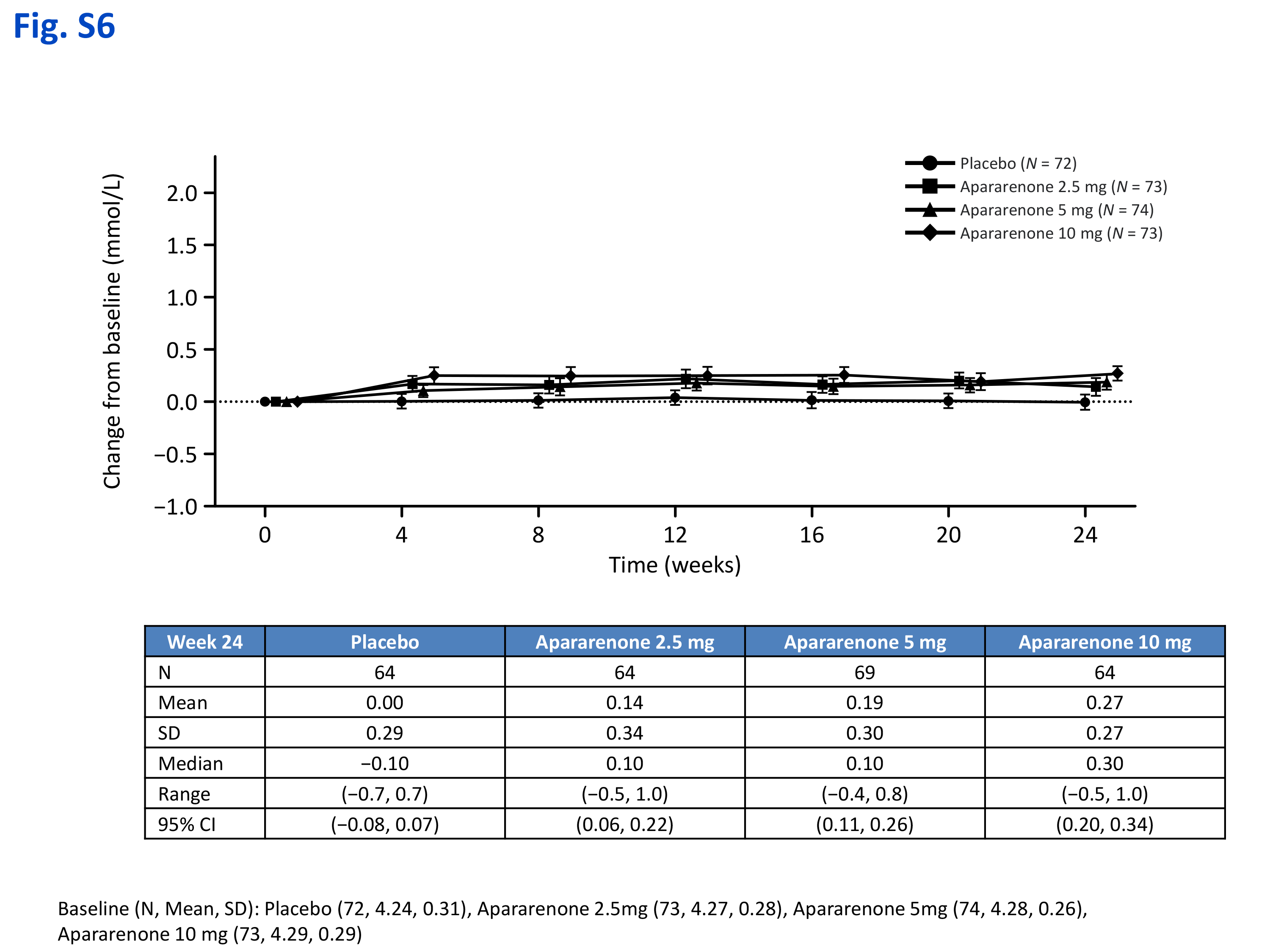

Supplement: Supplementary file 7 — Supplementary file7 (TIF 282 kb) [file 10157_2020_1963_MOESM7_ESM.tif]
